# Demographic overview of pilonidal sinus carcinoma: updated insights into the incidence

**DOI:** 10.1007/s00384-023-04344-6

**Published:** 2023-02-28

**Authors:** Mhd Firas Safadi, Marius Dettmer, Matthias Berger, Konstantinos Degiannis, Dirk Wilhelm, Dietrich Doll

**Affiliations:** 1 Department of Visceral, Thoracic and Vascular Surgery, University Hospital Carl Gustav Carus, Fetscherstraße 74, 01307 Dresden, Germany; 2 Vechtaer Institut für Forschungsförderung, VIFF e.V., Vechta, Germany; 3 Department of Trauma Surgery and Orthopedics, Reconstructive and Hand Surgery and Burn Medicine, German Armed Forces Central Hospital Koblenz, Koblenz, Germany; 4 Department of General and Visceral Surgery and Proctology, Diakoniekrankenhaus Chemnitzer Land, Hartmannsdorf, Germany; 5 Department of Trauma, Orthopedic and Hand Surgery, St. Marienhospital Vechta, Academic Teaching Hospital of the MHH Hannover, Vechta, Germany; 6 Faculty of Medicine, Clinic and Polyclinic of Surgery, Technical University Munich, Klinikum rechts der Isar, Munich, Germany; 7 Department of Procto-Surgery and Pilonidal Sinus, St. Marienhospital Vechta, Academic Teaching Hospital of the MHH Hannover, Vechta, Germany

**Keywords:** Pilonidal sinus, Pilonidal sinus carcinoma, Squamous cell carcinoma, Infection degeneration, Incidence, Pilonidal surgery

## Abstract

**Purpose:**

There are only rough estimates of the worldwide incidence of pilonidal sinus carcinoma. The purpose of the study is to explore the demographic characteristics of this disease and to provide more precise information about its incidence.

**Methods:**

The study included questioning the surgeons and pathologists in Germany in addition to a literature research. The literature investigation included all published articles about pilonidal carcinoma in all languages. The questionnaire included 1050 pathologists and all 834 hospitals with a surgical division in Germany. The outcome measures included the total number of cases, the language of publication, gender, age, country of origin, interval until the diagnosis of carcinoma, and reported incidence based on local studies.

**Results:**

From 1900 to 2022, we found 140 cases of pilonidal sinus carcinoma in 103 articles. The investigation revealed two additional unpublished cases from Germany. The male-to-female ratio was 7.75:1. The countries with the most cases were the USA (35 cases, 25.0%), Spain (13 cases, 9.3%), and Turkey (11 cases, 7.6%). The average age was 54.0 ± 11.8 years and the interval between the diagnosis of the disease and the development of carcinoma was 20.1 ± 14.1 years. There was a parallel increase in reported cases of pilonidal sinus disease and pilonidal carcinoma over the last century. The reported incidence varied from 0.03% to 5.56%. The worldwide calculated incidence equaled 0.17%.

**Conclusion:**

Due to underreporting and other causes, the incidence of carcinoma emerging on the background of pilonidal sinus disease is higher than reported.

**Supplementary Information:**

The online version contains supplementary material available at 10.1007/s00384-023-04344-6.

## Introduction

Cancer originating on the background of chronic pilonidal sinus disease (PSD) is extremely rare. The first case was reported by Heinrich Wolff in 1900, who described a 21-year-old woman with two recurrences after surgical treatment of pilonidal sinus. With no initial signs of malignancy, the specimen resected after the second recurrence showed squamous cell carcinoma [1]. Since then, only sporadic reports of PSD carcinoma were published from different countries, with about one published case per year worldwide. In 1944, Alford reported his results after having treated 86,333 patients with pilonidal disease in military hospitals. No cases of malignant transformation were documented [2].

The first authors aiming to estimate the incidence of PSD carcinoma (PSDCA) were Gaston and Wilde in 1965, who calculated an incidence of 0.11% (one PSDCA case out of *n* = 891 patients with PSD) [3]. This rate of around 0.1% was adopted in most subsequent publications as well as in surgical references and it has not been challenged since then.

With this research, we try to re-explore the worldwide incidence of PSD carcinoma based on the published reports. Since many PSDCA cases may not have been reported, we further assessed the presence of PSDCA in the surgical and pathological practice in Germany. Considering the worldwide increased incidence of primary pilonidal sinus disease [4–6], it is of interest to know if the reported increase in PSD incidence is also paralleled by an increase in PSDCA.

## Materials and methods

An online literature review was conducted using the databases provided by PubMed, Scopus, ScienceDirect, Medline, Cochrane, Web of science, and Google Scholar using the keywords “pilonidal AND sinus AND carcinoma; pilonidal AND carcinoma; pilonidal AND cancer; pilonidal AND tumor; pilonidal AND squamous” to find all articles related to PSDCA. Within each article or case report, we further analyzed the context and reference list to find articles that were missed or were otherwise not directly available in online databases such as historical reports, articles written before the creation of PubMed, and those that were published in languages other than English.

All articles reporting a pilonidal disease followed by the diagnosis of a malignant tumor in the pilonidal region without any previous cancer diagnosis were deemed adequate for further analysis regardless of the histological type of the tumor. Patients that had tumors without known pilonidal disease were judged as having primary skin neoplasms and were excluded from further analysis.

All non-English articles were translated and analyzed. The details of the reported cases were carefully examined and documented to exclude duplicated cases. As most of the publications contained a literature review, all cases from the literature were verified and cross-checked. Beyond the search for the raw numbers of reported PSDCA, we further scanned the literature for any information regarding the incidence of PSD carcinoma from centers around the world.

The number of published cases of PSDCA was compared to the total number of reported PSD patients. The comparison was based on the extended Pilonidal Sinus Disease database (courtesy of Stauffer et al.) [7] to obtain the total number of pilonidal cases published during the last 190 years since Mayo and Anderson [8, 9].

Since PSDCA is included in the category of cutaneous neoplasms, the German Cancer Registry does not have a distinct entry for the disease. To obtain information about unpublished cases, we contacted all 834 German hospitals that have a surgical division via post and email. Additionally, 1050 pathologists in Germany were contacted and asked if they had ever seen a histological specimen with PSDCA during their practice. All responses were crosschecked to exclude double reporting.

Datasets were extracted from the literature and organized into tables using Microsoft Excel (Microsoft ® Excel 2019, Version 16.0.11901.20170). The extracted data included the publication details (journal, year, and language of publication), the age and gender of the patients, the histological type of the tumor, the time interval between the diagnosis of PSD and PSDCA, and any available calculations of the incidence. Missing data were noted and the results were checked for plausibility. The analysis of the data was performed using pivot tables and the results were presented graphically using Microsoft Excel. The study was granted exemption from requiring ethics approval, as it solely analyses anonymized surveys from the surgeons and pathologists as well as anonymized statistical data from the literature. It neither contains nor discloses any patient-related information.

## Results

Over a period of 122 years (1900 to 2022), we found 140 published cases of PSD carcinoma in 103 articles (a table with all cases is available in the Online Resource). Of these 103 publications, six articles (5.8%), published between 1937 and 1968, could not be retrieved as full text from online or bibliotic resources despite an intensive search. Each of these six articles reported a single case and was cited with some additional details in later publications.

A total of 113 of 834 surgical departments (13,5%) and 110 of 1050 pathologists (10,5%) answered our questionnaire. The investigation yielded two additional, yet unpublished cases, adding up to a total of 142 cases. Since the details of these two unpublished cases are not documented in the literature, they were not included in the final review but were considered when discussing the incidence.

### Language and country of the publications

**Fig. 1:**
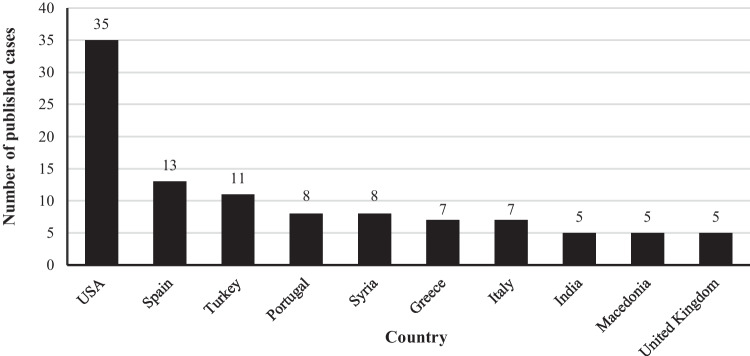
The ten most frequent countries with documented PSDCA

The vast majority of the articles (77/103; 74.8%) were published in English, with Spanish being the second most common language (6/103; 5.8%). Most articles (86/103; 83,5%) included a single case report, whereas the remaining articles reported multiple patients, ranging from 2 to 8 cases per article. The 140 cases were reported from 29 different countries, with the USA, Spain, and Turkey being the most prolific (Fig. 1).

Concerning the cases reported from Germany, only six patients were found over the study interval: two old cases published before 1940 [1, 10], two cases that were published recently [11, 12], and the two non-documented cases that were found through direct communication as mentioned above.

### The demographic characteristics of the patients

The majority of the population was male with 88.6% (124/140) versus 11.4% (16/140) being female (male-to-female ratio 1:7.75). The age of the patients at diagnosis of PSDCA ranged from 19 to 86 years (mean ± SD 54.0 ± 11.8 years; median 55.0 years). About 81.4% (*n* = 114) of the patients with PSDCA were between 40 and 69 years old. On average, the latency period between the first diagnosis of PSD and PSDCA was about 20.1 years (range 1 to 62 years, mean ± SD 20.1 ± 14.1 years; median 20.0 years).

### The histological type of the tumor

Squamous cell carcinoma was the most common entity reported with 128/140 cases (91.4%). Other types included basal cell carcinoma, mixed-type carcinoma, and adenocarcinoma. One case of rhabdomyosarcoma was reported in a series of 5 cases from Macedonia published in 1989 [13]. This case was never referred to in English literature.

### The number of published PSDCA cases

The total number of published PSDCA cases increased over time. More cases were published starting from 2000 and more than half of the cases (75/140, 53.6%) were published in the last 20 years.

**Fig. 2:**
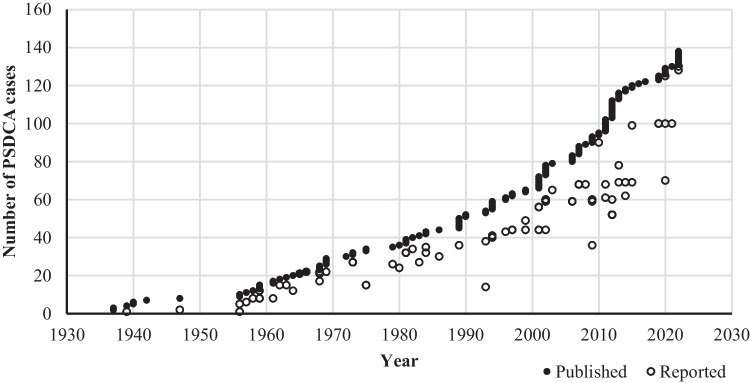
The stated number of PSDCA in the literature reviews (circles) compared with the real cumulative number of published PSDCA cases (dots) (reproduced with permission from Doll et al. [14])

In the majority of case reports, the authors performed a short literature review in an attempt to present an updated number of all published PSDCA cases. For each article, we checked how many previous cases were acknowledged (when available) and compared this to the actual published number of cases [14]. The results are plotted in Fig. 2, which shows a substantial underreporting of cases in most of the articles.

**Fig. 3:**
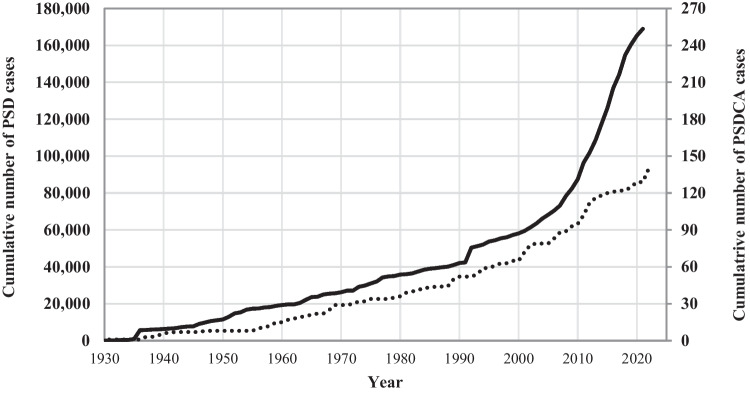
Comparison of cumulative cases of PSD (solid curve, presented on the left axis) and PSDCA (dashed curve, presented on the right axis) published over the last century

Figure 3 presents the cumulative number of published cases of PSD and PSDCA over time, based on the number of publications about PSD from the pilonidal sinus disease database [7]. The curves show a parallel increase in published cases for both PSD and PSDCA. Additionally, the interim period is also reflected in the chronological gap between the two curves.

### The incidence of PSDCA

In only *n* = 10 (9.7%) out of 103 publications about PSDCA, the authors did try to estimate the incidence of PSDCA according to the total number of pilonidal sinuses treated within their centers. The results are presented in Table 1, which shows the great variation in the calculated incidence between the studies, ranging from 0.03 to 5.56%. When considering all studies together, one can calculate a mean incidence of 0.17%.

**Table 1:** List of the publications that estimated the incidence of PSD carcinoma based on the total number of PSD cases treated in the relevant institute

**Study**	**Country**	**Year of publication**	**Period**	**Total years**	**PSD cases**	**PSDCA cases**	**Incidence**
Gaston and Wilde [3]	USA	1965	1940–1965	25	891	1	0.11%
Mukhadze [15]	Georgia	1975	-	-	245	2	0.82%
Pilipshen et al. [16]	USA	1981	1932–1981	49	2,457	2	0.08%
Gupta et al. [17]	India	1981	1965–1979	15	18	1	5.56%
Mirceva et al. [13]	Macedonia	1989	1967–1987	20	992	5	0.50%
Adamek et al. [18]	Czechia	1990	1970–1990	20	123	2	1.63%
Abboud and Ingea [19]	Lebanon	1999	-	-	500	1	0.20%
Velitchklov et al. [20]	Bulgaria	2001	1975–1999	24	3,734	1	0.03%
Alecha Gil et al. [21]	Spain	2006	1994–2004	10	367	3	0.82%
Alarcon-del Agua et al. [22]	Spain	2011	1995–2008	13	3,560	4	0.11%
**Total**					**12,887**	**22**	**0.17%**

*PSD* pilonidal sinus disease, *PSDCA* pilonidal sinus disease carcinoma

On the other hand, some authors performed a retrospective analysis of the histological reports of the resected pilonidal sinus in their centers and found no cases of carcinoma. Examples from the literature are shown in Table 2.

**Table 2:** List of the publications that could not detect any cases of PSDCA after a retrospective analysis of the histological reports in the relevant institute

**Study**	**Country**	**Year of publication**	**Period**	**Total years**	**PSD patients**
Casberg [23]	USA	1949	1941–1945	5	78,924
Boulanger et al. [24]	France	2018	2006–2014	9	731
Yüksel et al. [25]	Turkey	2020	2015–2019	4	905
Otutaha et al. [26]	New Zealand	2021	2010–2019	10	320

*PSD* pilonidal sinus disease

## Discussion

The incidence of carcinoma arising from pilonidal disease was classically estimated to be around 0.1%, although the precise incidence had never been investigated on a worldwide scale. The available data emerge from discrete single-center figures that appeared in case reports or small case series. For this reason, we aimed for summarizing the current evidence on this topic to bring all published reports together.

The internationally published data was complemented by seeking yet unpublished PSDCA cases in Germany by communicating with surgeons and pathologists and enquiring about their exposure to PSDCA. By this means, we discovered two additional PSDCA cases which had not been published and did not overlap with the existing literature. It is worth mentioning that our research group has recently identified other eight unreported PSDCA cases from Syria which were detected by questioning surgeons on social platforms. The results were published in a recent article [27]. Without this investigation, these cases would have never been detected and published.

Given that the total number of PSDCA cases published worldwide was 140 (including the eight newly-detected cases from Syria), and that two cases of unreported PSDCA were detected from Germany, we had to confess that there is a gap somewhere in the detection or reporting of malignant cases of PSD. Possible reasons are analyzed in the following paragraphs.

### Demographic characteristics

The data show that the PSDCA reports are not evenly distributed worldwide. As Asia and Africa have not reported major numbers of PSD patients [28–31], we expect only a few, if any, PSDCA patients from there.

As there is a marked 4:1 difference in male versus female pilonidal disease burden, we expected fewer PSDCA cases in female patients [30, 32, 33]. The review yielded a male/female ratio of nearly 8:1 for PSDCA. Women are less represented in PSDCA, perhaps because cancer most likely arises from a longstanding disease. Women are usually more health cautious than men [34] and they may have an earlier path to healing surgery, which would lead to a lower incidence of neoplastic malformation in comparison to men.

### Review of reports about the incidence of PSD carcinoma

Gaston and Wilde were the first to estimate the PSDCA incidence within their case report from 1965. After scanning all pathology records of Framingham Union Hospital and Boston City Hospital, they found specimens of pilonidal disease from 891 patients over 25 years. With only one PSDCA case per *n* = 891 PDS specimens, their calculated incidence was 0.11% [3].

To confirm their assumption, they compared their findings with the incidence of carcinomas arising on chronic wounds and fistulas outside of the sacral region. As reported by McAnally and Dockerty from Mayo Clinic [35], the rate of malignancy was “… about 0.23% in osteomyelitis sinuses, 0.1% in fistulas-in-ano, and 0.07% in chest empyema sinuses” [3]. Consequently, the calculated PSDCA incidence was consistent with other carcinomas of chronic inflammatory origin. This figure of 0.1% has since then been cited in nearly all reports about PSD carcinoma and until recent years.

Between 1965 and 2011, many authors who reported further cases of PSD carcinoma tried to shed more light on its incidence by examining the total number of cases treated in their institutes. A summary of the available information from the relevant studies is illustrated in Table 1. As the valued reader recognizes, the cohort sample sizes vary between 18 and 3734 patients. Interestingly, a reverse relationship between the size of the cohort and the incidence of carcinoma can be spotted: the larger the sample, the lower the calculated incidence. In his case series which included 4 carcinomas from a large patient cohort in 2001, Alarcon del Agua came close to the classical incidence of 0.11% [22].

As presented in Table 1, the lowest rates found in this study emerge from the USA, perhaps due to the better availability of healthcare services and the different awareness of well-being among the population. These rates may not reflect the probable higher incidence in chronically neglected cases, which reached 1.6% in the work of Adamek et al. from Czechia [18]. Surprisingly, one case of carcinoma was reported among only 18 PSD patients who presented to Banaras University Hospital in India within 15 years [17]. This would account for an outstanding rate of 5.6% for this institute. However, these results are most probably selection-biased: presumably, only a small number of “routine” PSD cases are being operated on in the said university hospital.

On the other hand, other researchers did not find any case of malignancy among large numbers of PSD patients. The classical publication of Casberg dates back to 1949, which reported 78,924 patients treated in military hospitals during World War II. Not a single case of malignant transformation was encountered in the whole group [23]. Milch et al. justified the findings by the fact that these patients were young soldiers with no neglected disease and not enough time for malignant transformation. The age factor, or to be more precise the duration of long-standing disease, plays an important role in the development of PSDCA [36]. As shown in Table 2, three other recent studies could not find malignant cases within PSD cohorts. However, the specimen size was underpowered to detect one PSD carcinoma within [14], as will be discussed below.

### Low incidence or underreporting?

So, why does the incidence of this malignant disease seem low compared with malignancies that emerge from chronic inflammatory lesions? We assume that the causes are divided into two categories:

(A) True low incidence of carcinogenesis due to:
  higher rate of early treatment and cure of the disease (especially in young patients, countries with developed health care, women, and body-conscious patients), or
  absence of squamous epithelium in many specimens upon histological examination (due to not yet epithelized fistula tracts in early disease).
(B) False low incidence due to underreporting due to:
  failure to submit the resected PSD tissue for histological examination,
  inadequate pathological examination for detection of microcarcinoma,
  use of minimally invasive approaches with no subsequent histological examination,
  the reluctance of surgeons to report the confirmed cases due to the dismal outcome,
  the disinterest of journals in accepting case reports for publications,
  publication of many articles in languages other than English, which makes them less findable,
  unavailability of some old publications in English as a full-text version, or
  publications before PubMed was established or outside of PubMed or similar libraries.

Concerning the first category, some researchers suggest that the development of carcinoma in pilonidal disease is less probable than in other inflammatory lesions. Most likely, PSD is not as longstanding and chronic and it is usually treated and cured in an early stage when the patient is still young [36]. As early as 1954, Davage showed that squamous epithelium is only found in about 50% of the resected pilonidal sinuses. In his opinion, the absence of epithelium delays carcinogenesis and cut the incidence to half [37].

On the other hand, many researchers stressed that PSD carcinoma is an underreported disease. Some resected pilonidal lesions may not be submitted for pathological examination [36], either due to the reluctance of the surgeon to let it be examined or for the sake of cutting costs, especially in developing countries where no insurance systems are available. Sometimes, the patient is deemed too young to have a PSDCA, so the pathological examination of the specimen is considered unnecessary [38]. Even when the specimen is submitted for examination, the pathologist may be reluctant to take enough sections from the apparently benign tissue, and hence fail to detect small foci of microscopic disease deep in the sinus tracts [14, 36]. As one pathologist said off record: “There is nothing less interesting than a PSD histology.”

Yüksel et al. drew attention to the increased use of minimally-invasive approaches for the treatment of pilonidal sinus, such as phenol obliteration, fibrin glue application, and video-assisted resection. In these cases, no biopsies are taken and no specimens are sent to pathology, which may regularly miss microscopic foci of malignant transformation [25].

Moreover, accidentally-discovered microscopic carcinoma will not be worth reporting, especially after a curative primary resection. The same applies to patients who do not survive the disease, as surgeons tend to publish successful cases and shelve those with bad outcomes [14, 39]. Moreover, many reputed journals, especially those listed in PubMed, no longer show interest in publishing case reports. Those who do will usually charge high publication fees that the authors are unwilling to pay for a case report, which contributes furthermore to the underreporting of cases.

Lastly, the evasion of small-of-interest case reports pushed more articles into the non-English literature, which was the case in about a quarter of all articles. It is not surprising that many of these were ignored in previous literature reviews [14, 39]. Even some papers written in English are not to be found in the current online libraries, which was the case with six old articles that were cited by other authors but could not be found online or in a printed version.

### What is the real incidence of pilonidal disease carcinoma?

Although many of the mentioned studies tried to estimate the incidence of PSD carcinoma, we question the accuracy of these estimations. Let us presume that the incidence is 0.1% or 1/1000. Based on the statistical estimations of the sample size for prevalence studies, the minimal sample size needed for achieving reasonable results with a presumed incidence of 1:1000 ± 0.25:1000 (error margin of 25%) would be at least 61,404 cases [40]. This required sample size far exceeds any sample size in the mentioned studies, with the only exception of the soldiers’ report, which involved young patients who are not representative of the usual PSD population.

Taking all documented cases together and accounting for all patients reported in Table 1, we arrive at a worldwide incidence of 0.17%. The total number of patients amounts to 12,887, which is still far less than the needed minimal sample size. However, this “total” incidence of 0.17 is about 1.5-fold the classical, often “copied” rate of 0.11% of PSDCA among all PSD patients. Due to the geographical variations, racial distribution, medical care availability, and patients’ compliance, variability of these rates is expected. For example, by omitting the results from the USA in Table 1, the incidence will jump to 0.2%.

As presented in the results section, we were able to detect two cases from Germany that had been left unpublished. Knowing that there were only four cases previously documented from Germany at all times, we may assume that there are at least 50% more cases than what is indeed reported. Taking this fact into account, we may raise the estimated rate further by 50%, thus yielding almost 0.3% in developing countries, if not even more, perhaps due to more unreported cases [27].

As the incidence rises with the duration of the disease, it is expected that the incidence will be lower if all patients were treated early. However, even in this case, it is unlikely that the incidence of PSDCA will fall to zero, as about 18,6% (26/140) of all carcinomas developed within 10 years of diagnosis and thus without a long duration of the disease.

### Is the incidence of pilonidal sinus carcinoma rising?

Most PSDCA were diagnosed in the last 20 years. As shown in Fig. 3, the cumulative numbers run nearly parallel to those of pilonidal disease. According to the above-made discussion and the presented literature, we estimate that the real incidence of PSD carcinoma should be at least 0.2%. One could speculate that the steep rise in the incidence of PSD should also result in a later increase in PSDCA. Nonetheless, the rise in treated cases could also reflect earlier and more comprehensive care of PSD patients, which will lead to a reduction in the PSDCA rate.

Accordingly, and in light of the current evidence, we believe that it is currently not possible to confirm whether the incidence is rising or not. However, our data show that the world has more cases of PSDCA than reported and that the incidence must be indeed higher than estimated. We hope that further large-scale studies and increased reporting will provide us with satisfying answers to this question.

## Conclusion

Every pilonidal sinus contains a certain degree of inflammation and infection, and even clinically inapparent PSD is considered an ongoing active disease. Early and curative treatment of every Pilonidal sinus is always recommended. While it may take an average of 20 years for neoplastic conversion from PSD to PSDCA to occur, this may happen in some patients in a much shorter period.

Accordingly, we recommend a comprehensive pathologic examination of all resected pilonidal specimens and not only of longstanding cases, recurrent disease, or elderly population. Although PSDCA is exquisitely rare, due to the increase in the incidence of pilonidal disease, we expect to encounter more patients with malignant transformation in the future. To identify the true incidence of PSDCA, we emphasize the importance of reporting and documenting all cases.

### Supplementary Information

Below is the link to the electronic supplementary material.

Supplementary file1 (PDF 479 KB)
