# Supplementary material for: Demographic overview of pilonidal sinus carcinoma: updated insights into the incidence
Source: Int J Colorectal Dis. 2023 Feb 28;38(1):56. doi: 10.1007/s00384-023-04344-6 (PMC9971075; doi:10.1007/s00384-023-04344-6)
Supplement: Supplementary file 1 — Supplementary file1 (PDF 479 KB) [file 384_2023_4344_MOESM1_ESM.pdf]

International Journal of Colorectal Disease

Demographic overview of pilonidal sinus carcinoma: Updated insights into the incidence

Mhd Firas Safadi, Marius Dettmer, Matthias Berger, Konstantinos Degiannis, Dirk Wilhelm, Dietrich Doll

Corresponding author: Mhd Firas Safadi, Department of Visceral, Thoracic and Vascular Surgery, University Hospital Carl Gustav Carus, Dresden, Germany
Email: doctor.safadi@gmail.com

Supplementary Material: Table of all documented cases of pilonidal sinus carcinoma
The following table lists all 140 reported cases of pilonidal sinus disease carcinoma in a total of 103 publications. The articles that reported more than one patient are presented on a corresponding number of rows.

Nr.	Year [a]	Authors	Country [b]	Gender	Age [c]	Interval [d]	Histology
1	1900	Wolff [1]	Germany	F	21	1	SCC
2	1937	Nosti [2]	Argentina	M	50	15	SCC
3	1937	Singleton [3]	N/A	M	55	N/A	N/A
4	1939	Schubert [4]	Germany	M	46	3	SCC
5	1940	Goldman and Kalow [5]	N/A	F	56	1	BCC
6	1940	Tendler [6]	USA	M	55	20	Adenoid
7	1942	Baraldi [7]	N/A	M	42	< 1	SCC
8	1947	Vara-Lopez [8]	Spain	F	67	40	SCC
9	1956	Hall and Lee [9]	USA	M	42	20	SCC
10	1956	Hayden [10]	UK	F	19	0	SCC/BCC
11	1957	Weinstein [11]	USA	M	43	21	SCC
12	1958	Matt [12]	USA	M	19	< 1	SCC/BCC
13	1959	Hibner and Cohn [13]	USA	M	55	24	SCC
14	1959	Hibner and Cohn [13]	USA	M	61	36	SCC
15	1959	Weinstein et al. [14]	USA	M	51	15	SCC
16	1961	Conole [15]	USA	M	62	38	SCC
17	1961	Terry et al. [16]	USA	M	53	26	SCC
18	1962	Boukalik and Salwan [17]	USA	M	61	43	SCC
19	1963	Milch et al. [18]	USA	M	68	3	SCC
20	1964	Cleveland and Green [19]	USA	M	32	7	SCC

Nr.	Year [a]	Authors	Country [b]	Gender	Age [c]	Interval [d]	Histology
21	1965	Gaston and Wilde [20]	Italy	M	86	51	SCC
22	1967	Marjani [21]	USA	F	55	20	BCC
23	1968	Brown and Rivera [22]	USA	F	78	62	SCC
24	1968	Critselis [23]	N/A	M	N/A	N/A	SCC
25	1968	Rubin et al. [24]	USA	M	72	25	BCC
26	1969	Boutet [25]	France	M	64	15	SCC
27	1969	Klevetenko [26]	Russia	M	49	19	SCC
28	1969	Klevetenko [26]	Russia	M	52	16	SCC
29	1969	Klevetenko [26]	Russia	M	42	16	SCC
30	1972	Yamada and Osaka [27]	Japan	M	49	20	SCC
31	1973	Puckett and Silver [28]	USA	M	46	27	SCC
32	1973	Puckett and Silver [28]	USA	M	55	25	SCC
33	1975	Mukhadze [29]	Georgia	M	36	5	SCC
34	1975	Mukhadze [29]	Georgia	M	23	< 1	BCC
35	1979	Lerner and Deitrick [30]	USA	M	48	10	SCC
36	1980	Golan et al. [31]	Israel	M	53	15	SCC
37	1981	Gupta et al. [32]	India	F	40	12	SCC
38	1981	Pilipshen et al. [33]	USA	M	62	35	SCC
39	1981	Pilipshen et al. [33]	USA	M	56	30	SCC
40	1982	Jamieson and Goode [34]	UK	M	63	20	SCC
41	1983	Anscombe and Isaacson [35]	UK	M	59	40	VCC
42	1984	Lineaweaver et al. [36]	USA	M	44	10	SCC
43	1984	Sagi et al. [37]	Israel	M	54	47	SCC
44	1986	Bark and Wilking [38]	Sweden	F	35	3	SCC
45	1989	Fasching et al. [39]	USA	M	48	20	SCC
46	1989	Mirceva et al. [40]	Macedonia	M	57	N/A	SCC
47	1989	Mirceva et al. [40]	Macedonia	M	57	N/A	SCC
48	1989	Mirceva et al. [40]	Macedonia	M	57	N/A	SCC
49	1989	Mirceva et al. [40]	Macedonia	F	57	N/A	SCC
50	1989	Mirceva et al. [40]	Macedonia	F	57	N/A	RMS
51	1990	Adamek et al. [41]	Czechia	M	45	4	SCC
52	1990	Adamek et al. [41]	Czechia	M	70	20	SCC
53	1993	Hoover et al. [42]	USA	M	34	10	VCC
54	1993	Kim and Thomas [43]	USA	M	63	44	SCC
55	1994	Davis et al. [44]	USA	M	44	10	SCC
56	1994	Davis et al. [44]	USA	F	60	10	SCC
57	1994	Davis et al. [44]	USA	M	53	30	SCC
58	1994	Jeddy [45]	UK	F	63	10	SCC

Nr.	Year [a]	Authors	Country [b]	Gender	Age [c]	Interval [d]	Histology
59	1994	Santanelli et al. [46]	Italy	M	40	20	SCC
60	1996	De Roos et al. [47]	Netherlands	M	63	44	SCC
61	1996	Kulyalat et al. [48]	USA	M	62	30	SCC
62	1997	Gur et al. [49]	Canada	M	54	10	SCC
63	1997	Gur et al. [49]	Canada	F	57	3	SCC
64	1999	Abboud et al. [50]	Lebanon	M	58	25	SCC
65	1999	Williamson et al. [51]	USA	M	59	22	SCC
66	2001	Borges et al. [52]	USA	M	32	N/A	VCC
67	2001	Borges et al. [52]	USA	M	51	10	SCC
68	2001	De Bree et al. [53]	Greece	M	48	25	SCC
69	2001	De Bree et al. [53]	Greece	M	59	30	SCC
70	2001	De Bree et al. [53]	Netherlands	M	67	5	SCC
71	2001	Pekmezci et al. [54]	Turkey	M	56	36	SCC
72	2001	Velitchklov et al. [55]	Bulgaria	M	51	24	SCC
73	2002	Adanali et al. [56]	Turkey	M	55	2	SCC
74	2002	Atmatzidis et al. [57]	Greece	M	61	30	SCC
75	2002	Cilingir et al. [58]	Turkey	M	42	16	SCC
76	2002	Cilingir et al. [58]	Turkey	M	70	15	SCC
77	2002	Jaime et al. [59]	Spain	M	58	20	VCC
78	2002	Matsushita et al. [60]	Japan	M	36	8	SCC
79	2003	Fernandez-Garcia et al. [61]	Spain	M	60	20	SCC
80	2006	Agir et al. [62]	Turkey	M	49	15	SCC
81	2006	Alecha Gil et al. [63]	Spain	M	54	N/A	SCC
82	2006	Alecha Gil et al. [63]	Spain	M	46	N/A	SCC
83	2006	Alecha Gil et al. [63]	Spain	M	48	N/A	SCC
84	2007	Frost et al. [64]	UK	F	80	59	SCC
85	2007	Kovacevic et al. [65]	Serbia	M	57	17	SCC
86	2007	Kovacevic et al. [65]	Serbia	M	49	21	SCC
87	2007	Malek et al. [66]	USA	F	40	2	SCC
88	2007	Mathew et al. [67]	India	M	N/A	N/A	SCC
89	2008	Mentes et al. [68]	Turkey	M	48	10	VCC
90	2009	Bolandparvaz et al. [69]	Iran	M	52	6	SCC
91	2009	Chatzis et al. [70]	Greece	M	50	15	VCC
92	2009	Sharma et al. [71]	India	M	52	20	SCC
93	2009	Tirone et al. [72]	Italy	M	45	16	SCC
94	2010	Galunic et al. [73]	Croatia	M	55	5	SCC
95	2010	Almeida-Gonçalves et al. [74]	Portugal	M	63	41	SCC
96	2011	Alarcon-Del Agua et al. [75]	Spain	M	57	12	SCC

Nr.	Year [a]	Authors	Country [b]	Gender	Age [c]	Interval [d]	Histology
97	2011	Alarcon-Del Agua et al. [75]	Spain	M	36	10	SCC
98	2011	Alarcon-Del Agua et al. [75]	Spain	M	62	40	SCC
99	2011	Alarcon-Del Agua et al. [75]	Spain	F	62	1	BCC
100	2011	De Martino et al. [76]	Italy	M	60	15	VCC
101	2011	Mello et al. [77]	Brazil	M	41	23	SCC
102	2011	Yetim et al. [78]	Turkey	M	52	20	SCC
103	2012	Almeida-Gonçalves [79]	Portugal	M	75	53	SCC
104	2012	Almeida-Gonçalves [79]	Portugal	M	51	33	SCC
105	2012	Almeida-Gonçalves [79]	Portugal	M	56	30	SCC
106	2012	Almeida-Gonçalves [79]	Portugal	M	56	25	SCC
107	2012	Almeida-Gonçalves [79]	Portugal	M	56	25	SCC
108	2012	Almeida-Gonçalves [79]	Portugal	M	56	25	SCC
109	2012	Almeida-Gonçalves [79]	Portugal	M	30	16	SCC
110	2012	Gaster et al. [80]	USA	M	64	N/A	SCC
111	2012	White et al. [81]	Australia	M	77	54	SCC
112	2012	Pandey et al. [82]	India	M	58	19	SCC
113	2013	Baykan et al. [83]	Turkey	M	49	20	SCC
114	2013	Goyal et al. [84]	India	M	70	21	SCC
115	2013	Nunes et al. [85]	Brazil	M	61	10	SCC
116	2013	Oruc et al. [86]	Turkey	M	52	10	SCC
117	2014	Cruz-Mendoza et al. [87]	Mexico	M	60	50	SCC
118	2014	Eryilmaz et al. [88]	Turkey	M	44	10	SCC
119	2015	Esposito et al. [89]	Italy	M	63	43	SCC
120	2015	Parpoudi et al. [90]	Greece	M	77	15	SCC
121	2016	Salih et al. [91]	Iraq	M	40	2	BCC
122	2017	Michalopoulos [92]	Greece	M	60	7	SCC
123	2019	Delvecchio [93]	Italy	M	83	30	SCC
124	2019	Ozkan [94]	Turkey	M	67	35	SCC
125	2019	Wronski [95]	Italy	M	58	30	SCC
126	2020	Mayol Oltra [96]	Spain	M	70	25	SCC
127	2020	Garcia [97]	Spain	M	49	17	SCC
128	2020	Lee [98]	USA	M	56	35	SCC
129	2020	Doll [99]	Germany	M	59	28	SCC
130	2021	Parajo [100]	Spain	M	53	20	SCC
131	2022	Dettmer [101]	Greece	M	61	25	SCC
132	2022	Safadi et al. [102]	Syria	M	50	5	SCC
133	2022	Safadi et al. [102]	Syria	M	55	2	SCC
134	2022	Safadi et al. [102]	Syria	M	60	N/A	SCC

Nr.	Year [a]	Authors	Country [b]	Gender	Age [c]	Interval [d]	Histology
135	2022	Safadi et al. [102]	Syria	M	45	10	SCC
136	2022	Safadi et al. [102]	Syria	M	60	N/A	SCC
137	2022	Safadi et al. [102]	Syria	M	60	3	N/A
138	2022	Safadi et al. [102]	Syria	M	60	20	SCC
139	2022	Safadi et al. [102]	Syria	M	52	1.5	SCC
140	2022	Pyon et al. [103]	USA	M	63	22	SCC

[a] Year of publication
[b] Patient's country of origin
[c] Age of the patient on the first diagnosis of PSDCA in years
[d] Interval between the diagnosis of PSD and PSDCA in years
Abbreviations: N/A, not available, F, female; M, male; BCC, basal cell carcinoma; SCC, squamous cell carcinoma; VCC, verrucous cell carcinoma; SCC/BSS, mixed type; RMS, Rhabdomyosarcoma

References

1. Wolff H (1900) Carcinoma auf dem Boden des Dermoids. Arch F Klin Chir Berl 62:731
2. Nosti R (1937) Cancer y cicatrices. An Cir Argentino 3:261-273
3. Singleton AQ (1937) Discussion of a paper on pilonidal sinus by M. Gage. Trans South Surg Assoc 50:71
4. Schubert H (1939) Karzinomatose Entartung von Steissdermoiden. Zentralbl Chir 66:2098
5. Goldman H, Kalow I (1940) Pilonidal cyst complicated by basal cell epithelioma. Bull Hosp Joint Dis 1:89
6. Tendler MJ (1941) Pilonidal sinus; a review of its literature and a report of 87 cases. South Med J 34:156
7. Baraldi A (1942) Degeneracion cancerosa de un Quiste Sacro-Coxigeo. Bol Soc Cir Rosario 9(103)
8. Vara-Lopez R (1947) Epithelioma of sacrococcygeal region following pilonidal cyst or pre-sacral teratoma; two cases. Rev Clin Espan 24:367-371
9. Hall A, Lee JG (1956) Squamous-cell carcinoma complicating a pilonidal sinus. Cancer 9(4):760-762
10. Hayden EP (1956) Medical Progress Proctology. NEJM 260(9):420-429
11. Weinstein M, Roberts M, Reynolds B (1957) Carcinoma complicating pilonidal sinus. N Y J Med 57(12):2089-2091
12. Matt JG (1958) Carcinomatous degeneration of pilonidal cysts: report of a case. DCR 1(5):353-355
13. Hibner R, Cohn R (1959) Squamous cell carcinoma arising in pilonidal sinus: report of two cases. Stanford Med Bull 17:198-203
14. Weinstein M, Roberts M, Reynolds B (1959) Pilonidal sinus carcinoma. JAMA 170(12):1394-1395
15. Conole FD (1961) Wide excision and primary closure in pilonidal disease: report of a case complicated by squamous cell carcinoma. DCR 4:435-438
16. Terry JL, Gaisford JC, Hanna DC (1961) Pilonidal sinus carcinoma. Am J Surg 102:465-469
17. Boukalik WF, Salwan FA (1962) Squamous cell carcinoma arising in a pilonidal sinus: case report. Ann Surg 156:157-160
18. Milch E, Berman L, McGregor JK (1963) Carcinoma complicating a pilonidal sinus. DCR 6(3):225-231

19. Cleveland BR, Green WO, Jr. (1964) Squarmous cell carcinoma arising in a pilonidal sinus. Surgery 55:381-386

20. Gaston EA, Wilde WL (1965) Epidermoid carcinoma arising in a pilonidal sinus. DCR 8(5):343-348

21. Marjani MA (1967) Basal cell epithelioma complicating a pilonidal sinus. Conn Med 31(2):106-108

22. Brown HW, Rivera J (1968) Epidermoid carcinoma arising in a pilonidal sinus. Report of a case and review of the literature. Int Surg 50(5):435-440

23. Critselis AN (1968) Squamous cell carcinoma in a pilonidal sinus (review of the literature and report of a case). Hellin Cheir 1:23-33

24. Rubin Z, Weinstein M, Hyman AB (1968) Basal cell epithelioma arising in a pilonidal sinus. Arch Dermatol 98(3):277-281

25. Boutet M (1969) [A case of epithelioma developed on a pilonidal sinus]. Laval Med 40(5):456-459

26. Klevetenko GI (1969) [Cancer developed from epithelial coccygeal sinuses (3 cases)]. Vopr Onkol 15(8):89-90

27. Yamada M, Osaka S (1972) [Case of squamous cell carcinoma arising in pilonidal cyst (sinus) or pyodermia chronica abscedens et suffodiens]. Nippon Hifuka Gakkai Zasshi 82(5):307-312

28. Puckett CL, Silver D (1973) Carcinoma developing in pilonidal sinus: report of two cases and review of the literature. Am Surg 39(3):151-155

29. Mukhadze GI (1975) Ozlokachestvlenie kist kresttsovo-kopchikovoĭ oblasti. Khirurgiia (2):140–141

30. Lerner HJ, Deitrick G (1979) Squamous-cell carcinoma of the pilonidal sinus: report of a case and review of the literature. J Surg Oncol 11(2):177-183

31. Golan J, Ashur H, Baruchin A, Ben-Hur N (1980) Squamous cell carcinoma arising in a pilonidal sinus. Int Surg 65(1):73-74

32. Gupta S, Kumar A, Khanna AK, Khanna S (1981) Pilonidal sinus epidermoid carcinoma: a clinicopathologic study and a collective review. Curr Surg 38(6):374-381

33. Pilipshen SJ, Gray G, Goldsmith E, Dineen P (1981) Carcinoma arising in pilonidal sinuses. Ann Surg 193(4):506-512

34. Jamieson NV, Goode TB (1982) Squamous cell carcinoma arising in a pilonidal sinus presenting with the formation of an abscess. Postgrad Med J 58(685):720-721

35. Anscombe AM, Isaacson P (1983) An unusual variant of squamous cell carcinoma (inverted verrucous carcinoma) arising in a pilonidal sinus. Histopathology 7(1):123-127

36. Lineaweaver WC, Brunson MB, Smith JF, Franzini DA, Rumley TO (1984) Squamous carcinoma arising in a pilonidal sinus. J Surg Oncol 27(4):239-242

37. Sagi A, Rosenberg L, Greiff M, Mahler D (1984) Squamous-cell carcinoma arising in a pilonidal sinus: a case report and review of the literature. J Dermatol Surg Oncol 10(3):210-212

38. Bark T, Wilking N (1986) Squamous-cell carcinoma in a pilonidal sinus. Case report. Acta Chir Scand 152:703-704

39. Fasching MC, Meland NB, Woods JE, Wolff BG (1989) Recurrent squamous-cell carcinoma arising in pilonidal sinus tract--multiple flap reconstructions. Report of a case. DCR 32(2):153-158

40. Mirceva D, Miskovski A, Boskovski L, Damevska L, Miskovska M, Dokic D, Daskalov O, Nikolova Z (1989) [Malignant changes in the pilonidal sinus]. Acta chirurgica iugoslavica 2:778-779

41. Adamek J, Antos F, Zeman V (1990) [Spinocellular carcinoma as a rare complication in pilonidal cyst]. Rozhl Chir 69(3):139-143

42. Hoover EL, Marrero R, Bumpers H, Coles M, Parsh S, Doerr R (1993) Surgical management of advanced squamous cell skin cancers. J Natl Med Assoc 85(12):912-915

43. Kim YA, Thomas I (1993) Metastatic squamous cell carcinoma arising in a pilonidal sinus. J Am Acad Dermatol 29:272-274

44. Davis KA, Mock CN, Versaci A, Lentrichia P (1994) Malignant degeneration of pilonidal cysts. Am Surg 60(3):200-204

45. Jeddy TA, Vowles RH, Southam JA (1994) Squamous cell carcinoma in a chronic pilonidal sinus. Br J Clin Pract 48(3):160-161

46. Santanelli F, Rubino C, Innocenzi D, Ribuffo D, Scuderi N (1994) Free flap failure in a patient with a long standing, infected, squamous cell carcinoma. Scand J Plast Reconstr Surg Hand Surg 28(4):305-308

47. de Roos KP, Koedam MI, Neumann HA (1996) [The non-healing ulcer; think of squamous cell carcinoma of the skin]. Ned Tijdschr Geneeskd 140(10):529-531

48. Kulaylat MN (1996) Multimodality treatment of squamouse cell carcinoma complicating pilonidal disease. Am Surg 62(11)

49. Gur E, Neligan PC, Shafir R, Reznick R, Cohen M, Shpitzer T (1997) Squamous cell carcinoma in perineal inflammatory disease. Ann Plast Surg 38(6):653-657

50. Abboud B, Ferran F, Chahine G (1999) Necrotizing fasciitis in sacrococcygeal pilonidal sinus in a patient with bone marrow aplasia. Treatment by large excision and closing by local flaps. Ann Chir Plast Esthet 44(5):552-555

51. Williamson JD, Silverman JF, Tafra L (1999) Fine-needle aspiration cytology of metastatic squamous-cell carcinoma arising in a pilonidal sinus, with literature review. Diagn Cytopathol 20(6):367-370

52. Borges VF, Keating JT, Nasser IA, Cooley TP, Greenberg HL, Dezube BJ (2001) Clinicopathologic characterization of squamous-cell carcinoma arising from pilonidal disease in association with condylomata acuminatum in HIV-infected patients. DCR 44(12):1873-1877

53. de Bree E, Zoetmulder FA, Christodoulakis M, Aleman BM, Tsiftsis DD (2001) Treatment of malignancy arising in pilonidal disease. Ann Surg Oncol 8(1):60-64

54. Pekmezci S, Hiz M, Saribeyoglu K, Akbilen D, Kapan M, Nasirov C, Tasci H (2001) Malignant degeneration: an unusual complication of pilonidal sinus disease. Eur J Surg 167(6):475-477

55. Velitchklov N, Vezdarova M, Losanoff J, Kjossev K, Katrov E (2001) A fatal case of carcinoma arising from a pilonidal sinus tract. Ulster Med J 70(1):61-63

56. Adanali G, Senen D, Tuncel A, Ibrahimoglu D, Erdogan B (2002) Squamous cell carcinoma developing in a pilonidal sinus. Plast Reconstr Surg 110(5):1367-1368

57. Atmatzidis K, Pavlidis T, Papaziogas B, Psaralexis K, Papaziogas T (2002) Squamous cell carcinoma arising in a neglected pilonidal sinus. Int J Col Dis 17(2):129-130

58. Cilingir M, Eroglu S, Karacaoglan N, Uysal A (2002) Squamous carcinoma arising from chronic pilonidal disease. Plast Reconstr Surg 110(4):1196-1198

59. Jaime SP, Julve JV, Alzamora MS, Santaló NB, Millán CV, Barón RE (2002) Malignant degeneration of pilonidal sinus: treatment. Clin Transl Oncol 4(6):331-334

60. Matsushita S, Ohtake N, Mochitomi Y, Fukumitsu K, Nishi M, Kanzaki T (2002) A case of squamous cell carcinoma arising in a pilonidal sinus. J Dermatol 29(11):757-758

61. Fernández-García LF, Blanco-Fernándeza G, Munuera-Romero L, Camposb A, Grau-Talens J, Vinagre-Velasco LM, Javier Téllez F (2003) Sinus pilonidal malignizado. Cir Esp 74(2):115-116

62. Agir H, Sen C, Cek D (2006) Squamous cell carcinoma arising adjacent to a recurrent pilonidal disease. Dermatol Surg 32(9):1174-1175

63. Alecha Gil J, Echenique-Elizondo M, Amondarain JA, Gorriz Arias G (2006) Epidermoid carcinoma arising in a pilonidal sinus. Cir Esp 80(1):53-56

64. Frost BM, Riddell AD, Austin S, Stephenson BM (2007) Malignancy in an old pilonidal sinus. Colorectal Dis 9(9):857

65. Kovacevic P, Visnjik N, Vukadinovic M, Kovacevic T, Mihajlovic D (2007) Carcinoma arising in pilonidal disease - report of two cases. Med Biol 14(3):133-137

66. Malek MM, Emanuel PO, Divino CM (2007) Malignant degeneration of pilonidal disease in an immunosuppressed patient: report of a case and review of the literature. DCR 50(9):1475-1477

67. Mathew J, Varghese S, Jagadeesh S (2007) The Limberg flap for cutaneous defects - a two year experience. Indian J Surg 69(5):184-186

68. Mentes O, Akbulut M, Bagci M (2008) Verrucous carcinoma (Buschke-Lowenstein) arising in a sacrococcygeal pilonidal sinus tract: report of a case. Langenbecks Arch Surg 393(1):111-114

69. Bolandparvaz S, Mohammadi AA, Riazi H, Monabbati A, Geramizadeh B (2009) Unusual presentation of squamous cell carcinoma on long-standing sacrococcygeal pilonidal sinus. Iran J Med Sci 34(2):149-151

70. Chatzis I, Noussios G, Katsourakis A, Chatzitheoklitos E (2009) Squamous cell carcinoma related to long standing pilonidal-disease. Eur J Dermatol 19(4):408-409

71. Sharma D, Pratap A, Ghosh A, Shukla VK (2009) Malignant transformation of a pilonidal sinus. Surgery 145(2):243-244

72. Tirone A, Gaggelli I, Francioli N, Venezia D, Vuolo G (2009) [Malignant degeneration of chronic pilonidal cyst. Case report]. Ann Ital Chir 80(5):407-409

73. Galunic R, Simic M, Igrec J, Galunic Bilic L, Brkljacic B, Srdoc (2010) Squamous carcinoma of the sacrum with pilonidal disease. Eurorad Case 8554

74. Almeida-Gonçalves JC, Ribeiro De Carvalho F (2010) Cryosurgery of advanced squamous-cell carcinoma arising in a sacrococcygeal pilonidal sinus report of a case and review of the literature. Skin Cancer 25(3):97-107

75. Alarcon-Del Agua I, Bernardos-Garcia C, Bustos-Jimenez M, Serrano-Borrero I, Casado-Maestre MD, Docobo-Durantez F (2011) Malignant degeneration in pilonidal disease. Cir Cir 79(4):346-350

76. De Martino C, Martino A, Cuccuru A, Pisapia A, Fatigati G (2011) Squamous-cell carcinoma and pilonidal sinus disease: Case report and review of literature. Annali Italiani di Chirurgia 82(6):511-514

77. Mello DF, Demario LA, Helene Jr A (2011) Squamous cell carcinoma as a late complication of sacrococcygeal pilonidal disease - case report. Rev Bras Coloproctologia 31(2):213-216

78. Yetim I, Semerci E, Özkan OV, Paltaci I, Diner G (2011) Squamous cell carcinoma arising in etiology of chronic pilonidal sinus disease: a case report. J Clin Anal Med 2(1):31-33

79. Almeida-Goncalves JC (2012) A curative cryosurgical technique for advanced cancer of sacrococcygeal pilonidal sinuses. J Surg Oncol 106(4):504-508

80. Gaster RS, Bhatt KA, Shelton AA, Lee GK (2012) Free transverse rectus abdominis myocutaneous flap reconstruction of a massive lumbosacral defect using superior gluteal artery perforator vessels. Microsurgery 32(5):388–392

81. White TJ, Cronin A, Lo MF, Huynh F, Donahoe SR, Lynch AC, Heriot AG (2012) Don't sit on chronic inflammation. ANZ J Surg 82(3):181-182

82. Pandey MK, Gupta P, Khanna AK (2014) Squamous cell carcinoma arising from pilonidal sinus. Int Woand J 11(4):354-356

83. Baykan H, Topuz Ö, Özyurt K, Cihan YB, Silay E (2013) Squamous cell carcinoma arised from chronic pilonidal sinus tract. Turk Plast Rekonstr Estetik Cerrahi Derg 21(1):43-44

84. Goyal S, Goyal S, Garg M (2013) Malignancy in pilonidal disease: Uncommon occurrence. Clin Can Inv J 2(2):153-155

85. Nunes LF, Castro Neto AK, Vasconcelos RA, Cajaraville F, Castilho J, Rezende JF, Noguera WS (2013) Carcinomatous degeneration of pilonidal cyst with sacrum destruction and invasion of the rectum. An Bras Dermatol 88(6 Suppl 1):59-62

86. Oruc M, Kankaya Y, Colak Aslan O, Ozer K, Kocer U (2013) Squamous cell carcinoma on the basis of pilonidal disease: A complication of chronicity. Eur J Plas Surg 36(9):605-606

87. Cruz-Mendoza I, Hernandez-Jesus B, Lopez-Perez E, Flores-Cobos AE, Garcia-Rodriguez F (2014) [Moderately differentiated squamous cell carcinoma associated pilonidal cyst]. Cir Cir 82(1):87-92

88. Eryilmaz R, Bilecik T, Okan I, Ozkan OV, Coskun A, Sahin M (2014) Recurrent squamous cell carcinoma arising in a neglected pilonidal sinus: report of a case and literature review. Int J Clin Exp Med 7(2):446-450

89. Esposito F, Lauro M, Tirone LP, Festa RM, Peluso G, Mazzoni G, Scognamiglio M, Grimaldi S, Fresini A (2015) Squamous cell carcinoma and pilonidal cyst disease. Ann Ital Chir 86(ePub)

90. Parpoudi SN, Kyziridis DS, Patridas D, Makrantonakis AN, Iosifidis P, Mantzoros IG, Tsalis KC (2015) Is histological examination necessary when excising a pilonidal cyst? Am J Case Rep 16:164-168

91. Salih AM, Kakamad FH, Rauf GM (2016) Basal cell carcinoma mimicking pilonidal sinus: A case report with literature review. Int J Surg Case Rep 28:121-123

92. Michalopoulos N, Sapalidis K, Laskou S, Triantafyllou E, Raptou G, Kesisoglou I (2017) Squamous cell carcinoma arising from chronic sacrococcygeal pilonidal disease: a case report. World J Surg Oncol 15(1):65

93. Delvecchio A, Laforgia R, Sederino MG, Minafra M, Carbotta G, Balducci G, Fabiano G, Fedele S, Palasciano N (2019) Squamous carcinoma in pilonidalis sinus: case report and review of literature. G Chir 40(1):70-74

94. Ozkan B, Cologlu H, Uysal CA, Ertas NM (2019) 35-year Onset of a Squamous Cell Carcinoma Originating from Sacral Pilonidal Sinus. PRS Global Open 7(12):e2553

95. Wronski K (2019) A rare case of squamous cell carcinoma arising from chronic sacrococcygeal pilonidal disease. Ann Ital Chir 8:1-3

96. Mayol Oltra A, Boldó Roda E, Lozoya Albacar R, Morillo Macias V, Nobleja Quiles N (2020) Squamous cell carcinoma over pilonidal chronic disease. A new therapeutic approach. Int J Surg Case Rep 70:172-177

97. García IC, Alcaide L, Saavedra MSZ, Pozo BM (2020) Squamous cancer originated in a recurrent pilonidal sinus: the progression of a benign and frequent condition to an infrequent cause of death. Rev Argent Coloproct 31:31-33

98. Lee H, Jehan F, Gachabayov M, Alizadeh K, Bergamaschi R (2020) Squamous cell carcinoma in untreated pilonidal cyst. Tech Coloproctol 25(2):241-243

99. Doll D, Koch M, Degiannis K, Wilhelm D (2020) Pilonidal Sinus Carcinoma foand in a Pilonidal Sinus – a surprise diagnosis from the pathologist. PSJ 6(1):21-25

100. Parajo A, Perez-Corbal L, Sanz JR, Seoane J, Vazquez-Garcia I, Cordoves I (2021) Squamous cell carcinoma related to chronic sacroccocygeal sinus: radical excision and reconstruction with a SGAP flap. Colorectal Dis 23(10):2788

101. Dettmer M, Bonni M, Degiannis K, Maak M, Doll D, Iesalnieks I (2022) Pilonidal sinus carcinoma dying from squamous cell carcinoma within 14 months after diagnosis. AJTES 6(1):979-981

102. Safadi MF, Ghareb K, Daher A, Dettmer M, Shamma H, Doll D (2022) Eight patients with pilonidal carcinoma in one decade—is the incidence rising? Cureus 14(7):e27054

103. Pyon RE, Mazumder A, Almajali F, Wong S (2022) Chronic pilonidal cyst with malignant transformation: a case report and literature review. Cureus 14(3)
